# The Fungal Protein Mes1 Is Required for Morphogenesis and Virulence in the Dimorphic Phytopathogen *Ustilago maydis*

**DOI:** 10.3390/jof8080759

**Published:** 2022-07-22

**Authors:** David Cánovas

**Affiliations:** Department of Genetics, Faculty of Biology, Universidad de Sevilla, 41012 Sevilla, Spain; davidc@us.es

**Keywords:** MesA, *Ustilago maydis*, polarized growth, dimorphic fungi, plant pathogen, morphogenesis, septin, actin

## Abstract

Polarized growth is a defining property of filamentous fungi, which plays an important role in different aspects of their biology, including virulence. However, little information is available about the determinants of cell surface organization and their role in polarized growth. The fungal protein MesA was identified in a genetic screen in *Aspergillus nidulans* and is involved in the stabilization of the polarity axes, but it has no evident role in budding yeast. In this work, I present evidence that in the dimorphic fungal phytopathogen *Ustilago maydis* MesA/Mes1 is involved in cell wall stability and polarized growth. *mes1* mutants were more sensitive to drugs provoking cell wall stress, and they displayed a temperature-sensitive phenotype. Actin cytoskeleton was disorganized in a *mes1* mutant, suggesting that there is a connection between Mes1, the actin cytoskeleton and polarized morphogenesis. The septin ring was also absent from the bud tip, but not the bud neck. Deletion of *mes1* provoked defects in endocytosis and vacuolar organization in the cells. Mes1 was essential for strong polarized growth in the hyphal form, but it was dispensable during low or moderate polarized growth in the yeast form in *U. maydis* at a permissive temperature. Consistently, *mes1* mutants showed delayed mating and they were avirulent.

## 1. Introduction

Fungal pathogens are an increasing threat to human life and an economical disaster for crop plantations. In many of these fungal pathogens, the infection process involves dramatic fungal morphological transformations before or during host invasion, and frequently these morphological transitions correlate with the ability to switch between isotropic and polar growth [1,2,3,4,5,6]. The ability to switch between different modes of growth is particularly clear in dimorphic fungi, which are able to produce yeast–hypha transitions in response to environmental conditions. Blocking the dimorphic transitions results in the loss of virulence [5,7]. Therefore, proteins that control dimorphic transitions likely play major roles in microbial adaptation to new environments and in pathogenicity, and consequently, it has become clear that the ability to modify the mode of growth may represent an “Achiles Heel” that can be exploited to limit fungal invasion of the host tissue.

Although yeast cells and hyphal filaments display different morphologies, only few genes have been identified that are specific to just one of the modes of growth. One example is the small GTPase Rac1/CflB, which is absent in yeast and only found in more complex fungi, and is able to grow in the filament form [8,9]. Another example seems to be the fungal protein MesA, which so far has been characterized in the filamentous fungi *Aspergillus nidulans* [10] and *Fusarium graminearum* [11]. MesA was identified in a genetic screen searching for mutants that enhance the formin *sepA* defects in *A. nidulans*, and is required for the stabilization of the polarity axis at the hyphal tip [10]. Homologs with weak homology were found in budding (*AFI1*) and fission (*SPBC0776.06c*) yeasts. However, deletion of those homologs displayed no obvious defects in budding yeast or modest defects in cell length and mating in fission yeast [10]. Avl9 was claimed to belong to the same superfamily as MesA, and it was identified in a genetic screen for mutants in the secretory pathway in *Saccharomyces cerevisiae* [12]. As is the case with Rac1-like proteins, putative homologues of MesA/Mes1 are present in dimorphic fungi, suggesting a role devoted to filamentous growth. Surprisingly, in spite of the apparent importance of the role of MesA-like proteins in filamentation, nothing is known about the role of these proteins in dimorphic fungi, where defects in yeast and filamentous growth can be compared in the same genetic background. In this study, I took advantage of the plant pathogen *Ustilago maydis,* a known model system, to study dimorphism and virulence [6,13,14] to characterize the roles that MesA-like proteins may have in pathogenic dimorphic fungi. *U. maydis* yeast cells grow by budding in the same way as *S. cerevisiae*, but they display an elongated cigar-shaped morphology [6], which makes them an excellent model to study MesA/Mes1 for comparative purposes.

*U. maydis* belongs to an important group of plant pathogens, the smut fungi, which can cause considerable grain yield loss and economic damage [15]. Pathogenic development in *U. maydis* initiates with the recognition of mating pheromone secreted by haploid cells of the opposite mating type on the plant surface [16]. After cell fusion, the combination of the cytoplasm of compatible cells leads to the formation of the *bE/bW* transcription factor that triggers growth of the *b*-dependent hyphae on the surface of plant epidermis [17]. On the plant surface, infectious hyphae grow in a polar manner by expanding at the apical tip and inserting regularly spaced septa at the distal pole, resulting in the formation of characteristic empty sections [18]. This growth mode enables the fungus to progress along the plant surface most likely to find an appropriate point of entry. Eventually, the filaments differentiate appressoria and penetrate the cuticule [19,20].

Our results here indicate that Mes1 is required for cell wall integrity and polarized cell growth at high temperatures. At normal to low temperatures, Mes1 plays an essential role during strong polarized growth in hyphae, but not during low or moderate polarized growth in the yeast form in *U. maydis*.

## 2. Materials and Methods

### 2.1. Strains and Growth Conditions

*U. maydis* strains are derived from FB1 or FB2 [21] and are listed in Table S1. Cells were grown at 28 °C unless otherwise indicated in complex medium (YP), complete medium (CM) or minimal medium (MM) [22] supplemented with 1% glucose (YPD, CMD or MMD) or 1% arabinose (YPA, CMA or MMA) as carbon sources. Expression of genes under the control of the inducible promoters *crg1* or *nar1* has been described previously [23,24]. Strains were grown in liquid media overnight and cultures were diluted to a final OD_600nm_ of 0.1. An amount of 30 µL was employed to inoculate YPD solid media in a 96-well plate and growth was monitored every hour for 24 h by using a plate reader, as previously reported [25].

### 2.2. Plasmid and Strain Construction

To produce a conditional *mes1^nar^* allele, I constructed a plasmid by ligation of two fragments into pNud1Nar digested with AflII and BamHI. The 5’ fragment (flanked by KpnI and AflII) was produced by PCR using the primers *mes1*-2 and *mes1*-8 (Table S2 contains the list of the primers used in this study). This fragment spans from nucleotide −1102 to nucleotide −20 (considering the adenine in the ATG as nucleotide +1). The 3’ fragment (flanked by BamHI and KpnI) was obtained by PCR amplification with primers *mes1*-4 and *mes1*-5 and spans from nucleotide +1 to nucleotide +1219. The resulting plasmid pMes1nar was integrated, after digestion with KpnI, by homologous recombination into the *mes1* locus replacing the native promoter by the *nar* promoter. Then, the expression of *mes1* was regulated by nitrogen source, being induced on nitrate and repressed on ammonium [23,24].

Deletion of *mes1* was carried out by homologous replacement following standard protocols [26,27]. Briefly, a pair of DNA fragments flanking the *mes1* ORF was amplified and ligated to a carboxin resistance cassette via SfiI sites. The 5′ fragment spans from nucleotide −1102 to nucleotide −100 (considering the adenine in the ATG as nucleotide +1), and it was produced by PCR amplification using the primers *mes1*-2 and *mes1*-9. The 3′ fragment spans from nucleotide +2773 to nucleotide +3808, and it was produced by PCR amplification using the primers *mes1*-10 and *mes1*-11.

After several attempts, deletion of *mes1* in FB2 could not be achieved by transformation of the deletion construct. Therefore, in order to isolate the mutant strain, cultures of UMD12 (FB1 ∆*mes1*) were mixed with the wild-type strain FB2 and the mixture was used to infect maize plants. Infection was allowed to develop until tumours were evident. Teliospores were obtained from the tumours and allowed to germinate and colonies were screened for the correct genotype.

Strains expressing *sep1-GFP*, *myo5-GFP* and *fim1-GFP* were constructed by homologous recombination in UMD7 (*a2 P_crg_:bW2 P_crg_:bE1 mes1^nar^*) under their respective native promoters following standard protocols [26,27], using plasmids previously described [28,29].

### 2.3. Light Microscopy and Image Analysis

Strains were incubated in the appropriate medium overnight to the exponential phase of growth. To induce *b*-dependent filamentation, AB33-derived cells were grown in the repressing medium CMD overnight, washed once in the inducing medium MMD-NO_3_, and finally resuspended in MMD-NO_3_ medium. To visualize SRDs or cell wall, cells were stained with 25 µg/mL filipin III (Sigma, St. Louis, MO, USA) for 4 min and washed twice with the culture medium or stained with calcofluor (Sigma), respectively. To visualize chitin deposition, cells were stained with wheat germ agglutinin-FITC (Sigma) for 10 min and washed twice with the culture medium.

To analyse endocytosis, cells were grown at the indicated temperature for 4 h and then stained with 16 µM FM4-64 (Molecular Probes) for 3 min in the dark. Then, cells were fixed with 1% (*v*/*v*) formaldehyde for 3–6 min and finally washed twice with culture medium. Alternatively, cells were pulsed for 2 min with 16 µM FM4-64 and then chased for 60 min in culture medium at the indicated temperature. 

*mes1^nar^* strains were grown in MMD-NO_3_ overnight to favour growth of the strain. Then, cells were washed once with YPD or CMD and incubated in YPD or CMD for ca. 5-6 h to repress the expression of *mes1* under the *nar* promoter. Appropriate controls of cells grown in MMD-NO_3_ were carried out in parallel. Induction of *b*-dependent filamentation in AB31-derived cells was performed similar to AB33 cells but employing complete media with arabinose (CMA).

Samples were visualized in a Nikon eclipse 90i microscope equipped with a Hamamatsu ORCA-ER CCD camera, and photographs were acquired using Metamorph software (Universal Imaging, Downingtown, PA, USA) and further processed with Adobe Photoshop 7.0 or using a Leica DMi8 microscope equipped with a Hamamatsu camera, and acquired using the Suite X (LAS X) software.

### 2.4. Transmission Electron Microscopy (TEM)

The wild-type strain FB1 and the ∆*mes1* strain UMD12 were grown overnight in YPD at 23 or 34 °C. Samples were washed twice with PBS buffer and then fixed with glutaraldehyde 2.5% in PBS buffer for 2 h at room temperature and post-fixed with 1% OsO_4_ in PBS buffer for 2 h at room temperature. Fixed samples were then dehydrated with increasing concentrations of ethanol for 5 min and finally with propylene oxide. Samples were embedded in Araldite resin. Thin sections were stained with uranil acetate and lead citrate. Images were acquired in a transmission electron microscope JEOL JEM1010 coupled to a Bioscan camera (Gatan) by using the software DigitalMicrograph 3.1. Images were further processed in Adobe Photoshop™ 7.0.

### 2.5. Mating and Plant Infections

To test for mating, strains were co-spotted on potato dextrose (PD) charcoal-containing plates and the plates were incubated at the indicated temperatures for 24 and 48 h, as previously described [24]. Confrontation assays were carried out as previously described [30].

Plant infections were performed as previously described [24] using the maize cultivar Early Golden bantam (Old Seeds, Madison, WI, USA) at 25 °C to allow growth of the ∆*mes1* strains.

### 2.6. Bioinformatic Analyses

OrthoFinder version 2.2.0 [31] with default options was used to assess orthology among the fungal proteomes, as previously reported [32]. The tree was drawn in iTOL [33]. Protein domains were annotated employing the NCBI web Cd-search tool [34] against the CDD database and in fungidb (https://fungidb.org/fungidb/app, accessed on 23 March 2022) [35]. Potential transmembrane domains were identified using DAS [36].

## 3. Results

### 3.1. The MesA/Mes1 Homologue in U. maydis

*A. nidulans* MesA and *F. graminearum* Mes1 protein sequences were employed as baits to carry out a BLAST search against *U. maydis* genomic sequence data. One single hit was found, corresponding to the predicted protein UMAG_03261 in an annotated *U. maydis* genome database [37], which is currently available in fungidb (https://fungidb.org/fungidb/app/record/gene/UMAG_03261, accessed on 23 March 2022). This protein was named Mes1, according to the standard nomenclature for this organism. Comparison of the predicted protein sequences revealed that umMes1 is more similar to *F. graminearum* Mes1 (*e*-value = 5.3 *×* 10^−116^) than to *A. nidulans* MesA (*e*-value = 8.9 *×* 10^−86^). Two to three potential transmembrane domains were identified using DAS [36] in positions 385–394, 411–428 and 748–756, differing from the location of the two potential transmembrane domains in anMesA. However, no potential transmembrane domains were predicted using TMHMM available at fungidb [35]. A search for conserved domains found homology to Afi1 (Avl9 superfamily, pfam07792) and SPA (Stabilization of Polarity Axis, pfam08616).

**Figure 1:**
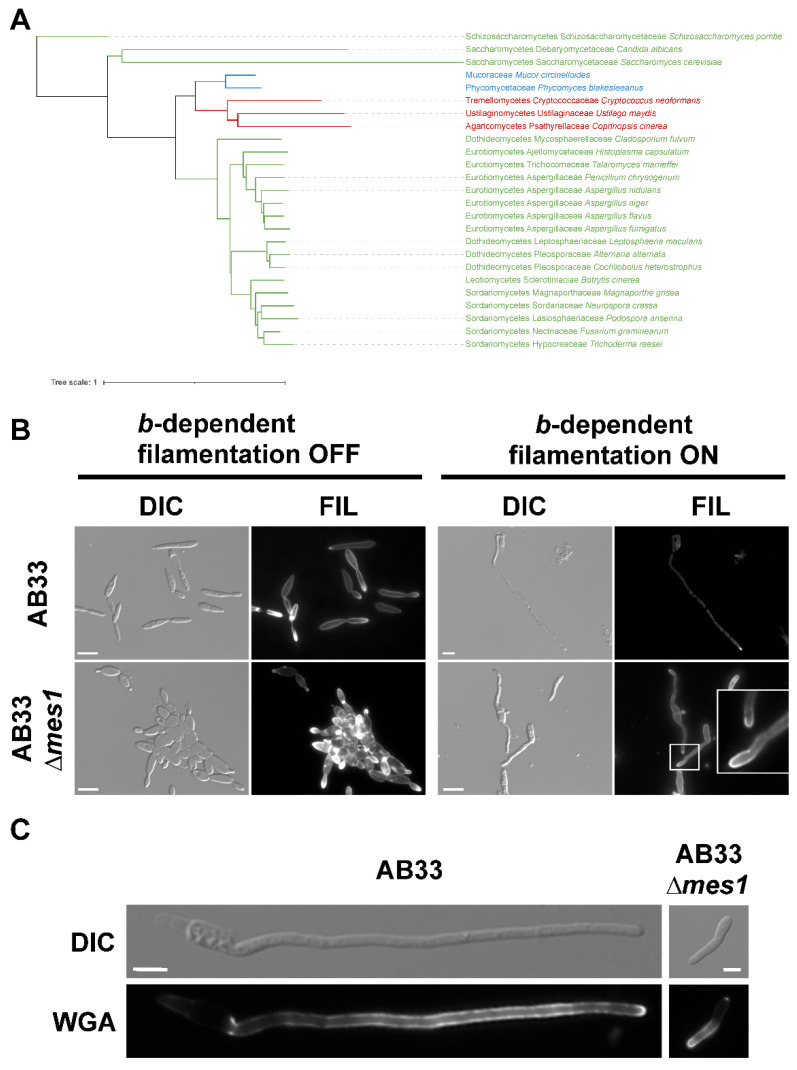
Role of Mes1 in filamentation. (**A**) Phylogenetic tree depicting the evolutionary relationship between the MesA/Mes1 homologs of yeast, filamentous and dimorphic fungi. Green: species belonging to Ascomycota; Red: species belonging to Basidiomycota; Blue: species belonging to Mucoromycota. (**B**) AB33 and AB33 ∆*mes1* strains were grown in MM-NO_3_^−^ to induce filamentation (“*b*-dpendent filamentation ON”) or in CMD to observe yeast cells (“*b*-dpendent filamentation OFF”) for 7 h at 28 °C. Strains were stained with filipin to observe SRDs. Bar size = 10 µM. (**C**) Filaments of AB33 and AB33 ∆*mes1* were stained with WGA-FITC to observe chitin deposition. Bar size = 5 µM.

In order to perform phylogenetic analyses, orthogroups were created by whole-genome comparisons of selected species from different clades belonging to ascomycetes, basidiomycetes and zygomycetes, as previously reported [32]. Only one homolog of MesA/Mes1 was found in each of the species selected in this study (Figure 1A). As previously reported and strikingly, *U. maydis* Mes1 cluster together with other homologs of other filamentous fungi belonging to the ascomycetes, basidiomycetes and zygomycetes, and are well separated from homologs of budding and fission yeasts. The homologs from other dimorphic fungi, such as *Cryptococcus neoformans*, *Talaromyces marneffei* and *Hisoplasma capsulatum* also clustered together with the homologs of filamentous fungi, with the exception of the *Candida albicans* homolog, which clustered together with *S. cerevisiae* Afi1 (Figure 1A). Yeast Avl9p was not found in this orthogroup.

### 3.2. Mes1 Is Required for Full Filamentous Growth in U. maydis

Previous characterization of the MesA/Mes1 proteins in *A. nidulans* and *F. graminearum* revealed that it is required for the stabilization of polarity axes in the filament [10,11]. In *U. maydis*, the formation of the infective filament can be mimicked in vitro using the AB33 strain [23]. This haploid laboratory strain carries the compatible *bW1/bE2* genes under control of the nitrate-inducible *nar1* promoter [23]. Thus, *b*-dependent infective filament formation can be elicited in AB33 by changing the nitrogen source. The whole *mes1* coding region was deleted in the AB33 strain. Induction of *bW2* and *bE1* expression in AB33 cells for about 6 h resulted in the formation of a filament (around 90 µm long). In this filament, it was possible to observe the formation of empty cell compartments at the basal cell end. In contrast, AB33 ∆*mes1* cells shifted to inducing conditions for 6 h at 28 °C were able to put up a short filament that was incapable of further elongation (Figure 1B,C). Staining with FITC-labelled wheat germ agglutinin (WGA) displays active sites of chitin deposition, while filipin binds to sterols in the membrane and stains sterol-rich domains (SRDs) [23,38,39,40,41]. The wild type displayed discrete patches of both WGA and filipin staining at the hyphal tip. In AB33 ∆*mes1* cells, although they displayed shorter filament cells, they also accumulated discrete patches of WGA and filipin staining at the tips (Figure 1B,C). 

### 3.3. Mes1 Is Required for Yeast Growth in U. maydis

**Figure 2:**
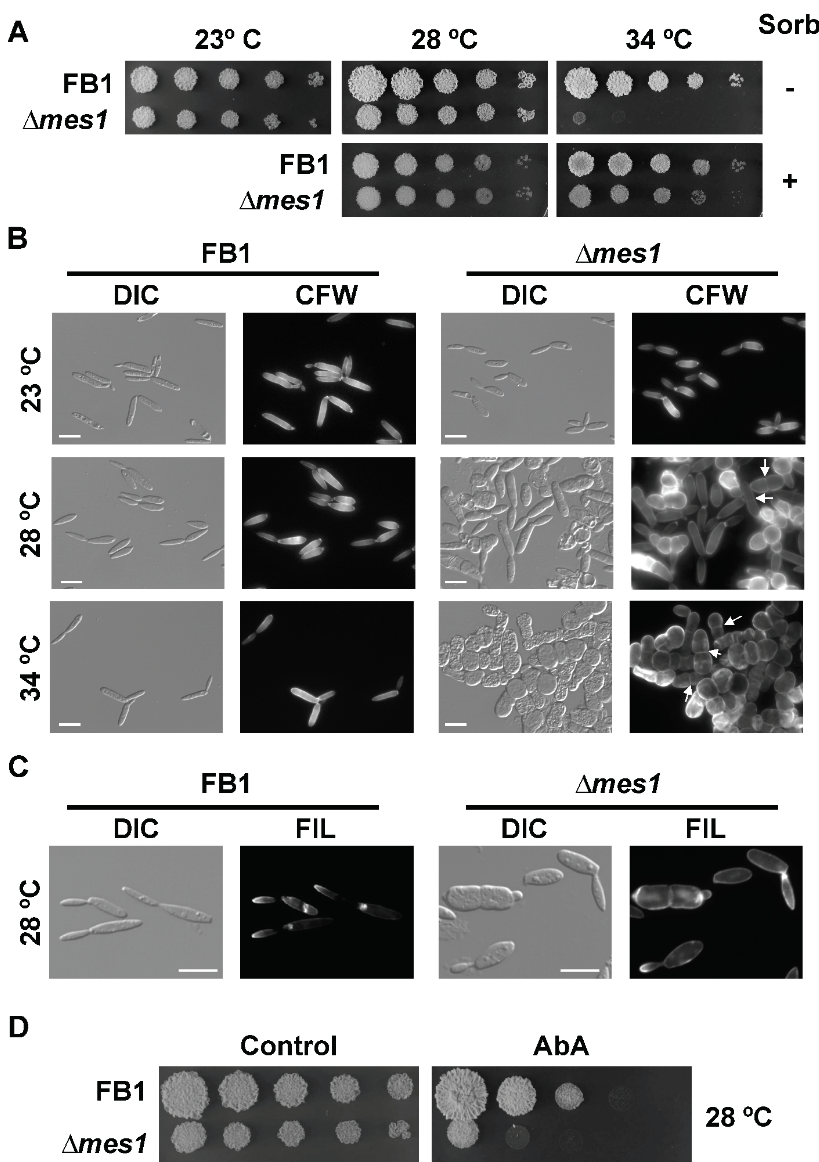
∆*mes1* loss of polarity is temperature-dependent. (**A**) Ten-fold serial dilutions were spotted on YPD plates with (+) or without (−) 1 M sorbitol. Plates incubated for 3 days at the indicated temperatures and photographed. (**B**) FB1 and FB1 ∆*mes1* strain were grown in YPD medium to the exponential phase of growth, diluted into fresh YPD medium and grown overnight at the indicated temperatures. Cells were stained with CFW and visualized under the microscope. The loss of polar growth in the ∆*mes1* strain is characterized by the roundish cell shape, loss of the budding division, and the placing of cross medial septa. Some cells showed abnormal chitin deposition. Arrows point to some examples of cross medial septa. Bar size = 10 µM. (**C**) FB1 wild-type and ∆*mes1* strains were grown in CMD to the exponential phase of growth and stained with filipin as described in the Materials and Methods section. Filipin staining was localized in the tips of wild-type and mutant strains. Bar size = 10 µm. (**D**) Cultures of FB1 and FB1 ∆*mes1* strains were grown to an OD_600_ about 1.0 and 10-fold serial dilutions were spotted on YPD plates containing 0.1 µg/mL aureobasidin A (AbA).

In contrast to what happens in yeasts, deletion of *mes1* in the AB33 strain unexpectedly produced yeast cells with morphological defects under non-inducing conditions of filamentation (see Figure 1A). To faithfully address whether Mes1 has any role in the growth of *U. maydis* yeast cells, the deletion mutant was reconstructed in the haploid FB1 strain. The resulting strain was viable, although it appears to be thermosensitive. No difference in growth on solid medium was observed when cells were incubated at the interval between 23–28 °C, but the mutant strain was unable to develop colonies when incubated at 34 °C (Figure 2A). This defect was remediable with the addition of 1 M sorbitol to the media, suggesting defects in the cell wall of ∆*mes1* strains. The colony size of the ∆*mes1* strain was smaller than the wild type, even at the permissive temperatures. Using a method developed to monitor fungal growth on solid media, it could be observed that the growth of the deletion strain was indeed slower than the growth of the wild-type strain (Figure S1).

I analysed the effects of the absence of Mes1 in yeast cells grown in liquid cultures. FB1 wild-type and ∆*mes1* strains were grown in YPD at the indicated temperature (Figure 2B). ∆*mes1* cells displayed a nearly wild-type morphology at 23 °C. The morphological defects were enhanced with increasing temperature, showing an intermediate phenotype at 28 °C. At the highest temperature tested, 34 °C, ∆*mes1* cells appeared as rows of stunted cells. Mutant cells have stopped growth, became round and are unable of budding division. Division appeared to proceed by placing a cross septa in the middle region of the cell.

Supplementation of liquid media with 1 M sorbitol alleviated the apparent cell wall defects by allowing growth of the deletion mutant at 34 °C. However, sorbitol could not remediate the morphological and polarity defects of the mutant (Figure S2). 

A conditional knock-out of *U. maydis mes1* was constructed, in which the expression of *mes1* could be induced with nitrate or repressed with ammonium (or in complex media). FB1 *mes1^nar^* recapitulated the phenotype of the ∆*mes1* deletion strain: it could grow on YPD + 1 M sorbitol at 34 °C, but there was no growth in YPD solid media at 34 °C (Figure S3). Furthermore, expression of the *mes1* conditional allele on media containing nitrate allowed the growth of the *mes1^nar^* strain at 34 °C, which supports the above observations that the lack of *mes1* renders the cells thermosensitive.

Since yeast cells displayed drastic morphological defects at 34 °C and an intermediate phenotype at 28 °C, one possibility was that this temperature was also affecting filament formation (as seen in Figure 1B,C). In order to test this hypothesis, filamentation was induced at 23 °C (Figure S4). In line with what happened at 28 °C, most of the mutant cells were incapable of forming a filament, and only some cells were able to put up filaments that were drastically shorter than the wild-type filaments, suggesting that mutation of *mes1* provokes two distinguishable phenotypes: cell wall defects and polarized growth defects.

The above morphological defects observed in *mes1*-defective cells were consistent with problems in the ability to achieve polarized growth. In fungi, membrane microdomains rich in sphingolipids and sterols (the so-called ‘Sterol-Rich Domains’, SRD) are thought to play an essential role during polarized growth [38,42]. Moreover, the localization of SRDs was dramatically altered in *A. nidulans mesA1* mutant [10] and slightly affected in *F. graminearum* ∆*mes1* [11]. Therefore, in order to assess the distribution of SRDs in Mes1-defective cells, cultures of wild-type and mutant cells were stained with the sterol-binding compound filipin. Using fluorescence microscopy, filipin staining could be still visualized at the tips of the cells and buds (Figure 2C). However, some cells showed a fainter staining signal, suggesting that they could be partially affected in the organization of membrane domains. Because this defect was very subtle, I wondered whether ∆*mes1* cells were more sensitive to compounds affecting the synthesis of sphingolipids, such as aureobasidin A [38,43]. In fact, that was the case (Figure 2D), suggesting that, as is the case in *A. nidulans* and *F. graminearum*, SRDs may be affected in *U. maydis*.

### 3.4. Deletion of mes1 Affects the Actin Cytoskeleton

**Figure 3:**
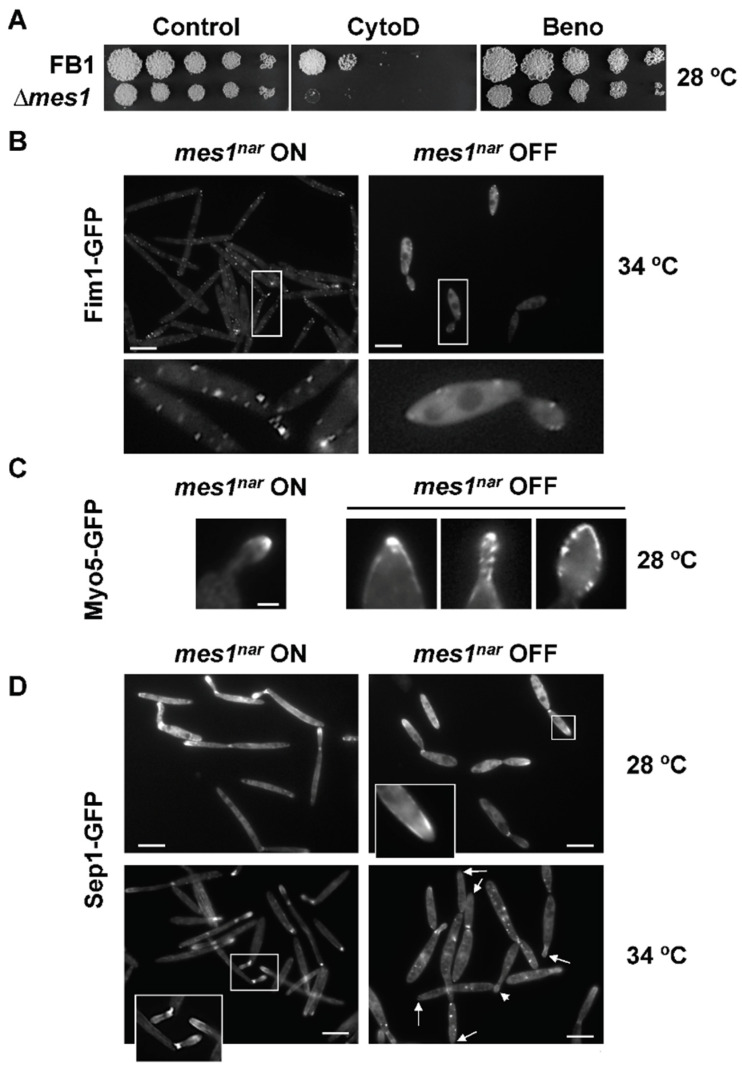
Mes1 and the actin cytoskeleton. (**A**) FB1 wild-type and FB1 ∆*mes1* strains were inoculated onto YPD plates containing 10 µM cytochalasin D (CytoD) or 1 µM benomyl (Beno), which disrupt actin cables or microtubules, respectively. ∆*mes1* strain was more sensitive to cytochalasin D but not to benomyl. AB31 *mes1^nar^* strains harbouring a Fim1-GFP (**B**), Myo5-GFP (**C**), or Sep1-GFP (**D**) fusion were grown under inducing (*mes1^nar^* ON) or repressing (*mes1^nar^* OFF) conditions for *mes1* at the indicated temperature. GFP fluorescence was visualized under the microscope. Lower panels in (**B**) or insets in (**D**) represent magnifications of the indicated areas. Bar size = 2 µm.

In *A. nidulans,* MesA recruits the formin SepA, facilitating the assembly of actin cables [10]. In order to investigate a putative connection between Mes1 and the cytoskeleton in *U. maydis*, dilutions of the wild-type and the ∆*mes1* strains were spotted onto YPD plates containing cytochalasin D, a well-known actin inhibitor drug, or benomyl, a microtubule destabilizer drug (Figure 3A). The deletion strain was clearly more sensitive to cytochalasin D, but not to benomyl, suggesting an actin-based defect. 

The increased sensitivity to cytochalasin D and the thermosensitivity of the ∆*mes1* strain suggested a putative connection between Mes1 and the actin cytoskeleton in *U. maydis*. To visualize the actin cytoskeleton, I employed a fimbrin-GFP fusion to tag F-actin in patches [29] and a Myo5-GFP fusion to analyse the functionality of the actin cables [44]. It is known that heat shock promotes depolarization of the actin cytoskeleton in *S. cerevisiae* [45]. By using a wild-type strain expressing a Fim1-GFP fusion, it was observed that, although the stability of the actin patches was also temperature-dependent in *U. maydis* (Cánovas D., Pérez-Martín, J. Unpublished Work), incubation of the Fim1-GFP strain at 34 °C did not result in the disassembly of the actin patches (Figure 3B).

To investigate the role of *mes1* in the stability of the actin cytoskeleton, the *U. maydis mes1* conditional knock-out strains were employed, in which the expression of *mes1* was induced (nitrate) or repressed (ammonium) for 5–6 h before analysis, as described in the Materials and Methods section. When *mes1* expression was repressed at 34 °C, cells were shorter compared to the control grown under the same conditions, and actin patches were drastically reduced or nearly absent, suggesting that Mes1 may facilitate the assembly or that it contributes to the stabilization of actin patches at the cell surface (Figure 3B). This points to a putative Mes1 activity in either the assembly or the maintenance of the actin cytoskeleton at high temperatures. 

In a Mes1-depleted strain, Myo5-GFP could be still visualized at the tips at 28 °C. However, cells showed the localization of Myo5-GFP spread out in the bud (Figure 3C). Increasing the temperature to 34 °C resulted in a major delocalization of Myo5-GFP, even in the presence of Mes1, and therefore it could not be tested.

Septins are scaffolds necessary for the localization of factors involved in F-actin re-modelling, polarized growth and cell division, and they play a crucial role in determining the cell shape [46]. A Sep1-GFP fusion was employed as an indicator of the septin-rings status in the *mes1* conditional mutant (*mes1^nar^*). At 28 °C, Sep1-GFP localized to the bud neck and as a band-like structure just behind the tip (Figure 3D), as previously reported [28]. At 34 °C, Sep1-GFP localization was not altered in the wild-type strain. In the *mes1^nar^* strain under repression conditions, Sep1-GFP still localized to the bud neck, but it was mostly absent from the tip of the bud (Figure 3D). Additionally, mutant strains frequently displayed disperse patches of fluorescence in the cells grown at 34 °C, unlike the wild-type strain. 

### 3.5. The ∆mes1 Strain Is More Sensitive to Membrane and Cell Wall Disruptants

**Figure 4:**
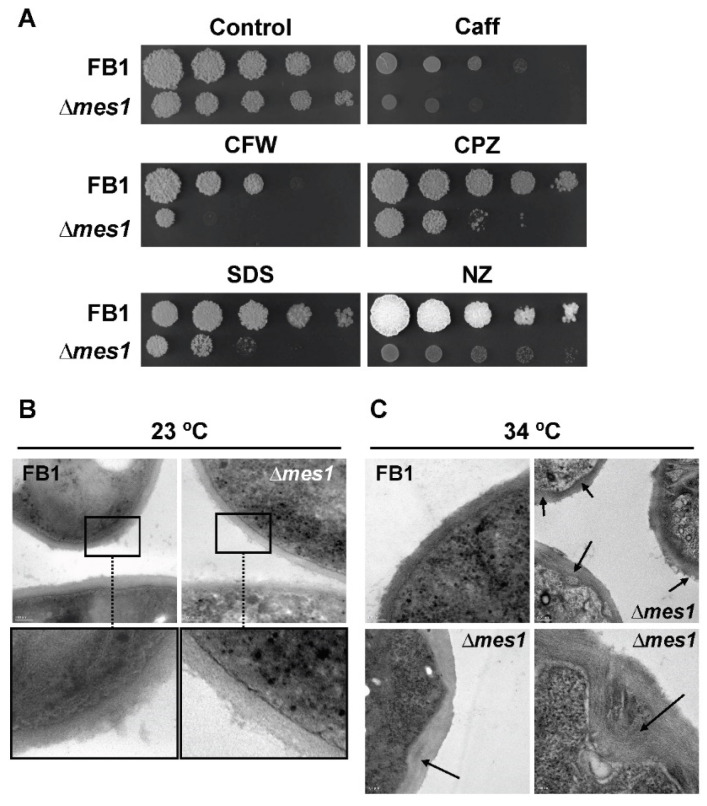
Mes1 is required for cell surface stability. (**A**) ∆*mes1* strain shows increased sensitivity of membrane and cell wall disruptants. Cultures of FB1 and FB1 ∆*mes1* strains were grown to an OD_600_ about 1.0 and 10-fold serial dilutions were spotted on YPD plates containing 1.5 mM caffeine (Caff), 50 µM calcolfluor white (CFW), 50 µM chlorpromacyn (CPZ), 0.0075% SDS, or 1 µM Nikkomycin Z (NZ). Plates were incubated at 28 °C and photographed. (**B**,**C**) TEM of ∆*mes1* strain. FB1 and FB1 ∆*mes1* strain were grown in YPD medium to the exponential phase of growth at room temperature, diluted into fresh YPD medium and grown overnight at 23 °C (**B**) or 34 °C (**C**). Samples were fixed and prepared for TEM. Note that differences were more dramatic at 34 °C. The cell surface of the ∆*mes1* strain appeared as a set of unstructured layers, with frequent invaginations (marked with arrows). Lower panels in (**B**) displayed computer-assisted magnifications of the indicated area.

A functional *mes1* gene was necessary for growth at a high temperature (34 °C). This defect was remediable with the addition of 1 M sorbitol to the media, suggesting defects in the cell wall of ∆*mes1* strains. In order to gain information at this respect, the FB1 ∆*mes1* strain was tested for increased sensitivity to several drugs affecting membrane and cell wall integrity (Figure 4A). Caffeine (Caff) produces stress by affecting the signal transduction cascades controlling cell wall assembly [47]. Calcofluor white (CFW) binds to chitin and interferes with normal cell wall assembly [48,49]. Chlorpromazine (CPZ), a cationic amphipath, induces plasma membrane stretch and produces depolarization of the actin cytoskeleton [45]. Sodium dodecyl sulfate (SDS) is a detergent that depolarizes the actin cytoskeleton in *S. cerevisiae* [45]. Nikkomycin Z (NZ) is a competitive inhibitor of chitin synthases [50,51]. Surprisingly, FB1 ∆*mes1* strain was more sensitive than the wild-type strain to all of the tested drugs, affecting either cell wall or the plasma membrane, which suggests that the deletion of the *mes1* gene had pleiotropic effects on the integrity of the plasma membrane and cell wall in *U. maydis*.

To obtain further insight into the cell surface defects of the *mes1* deletion strain, FB1 wild-type and ∆*mes1* were grown overnight at both 23 °C and 34 °C. Cells were processed and prepared for TEM as described in Materials and methods. At 23 °C, little differences could be observed between wild-type and mutant cells (Figure 4B). The inner layer appeared thinner and more dense to the electrons in the mutant strain, while the outer layer looked more loose. As expected, the differences were more dramatic at 34 °C (Figure 4C). The wild-type strain showed two electron-dense layers and an outer loose layer. The inner dense layer was nearly not discernible in the ∆*mes1* mutant strain. The outer layer was less dense to the electrons and thicker than in the wild-type strain, and lacked the surface loose layer, suggesting that the cell wall was unstructured in the deletion strain. In addition, the mutant strain displayed an irregular contour with invaginations of different size and degrees that could be frequently observed in the surface of the ∆*mes1* strain. These observations further suggest that Mes1 participates in the stability of the plasma membrane and cell wall components in *U. maydis*.

### 3.6. Endocytosis in the ∆mes1 Mutant

**Figure 5:**
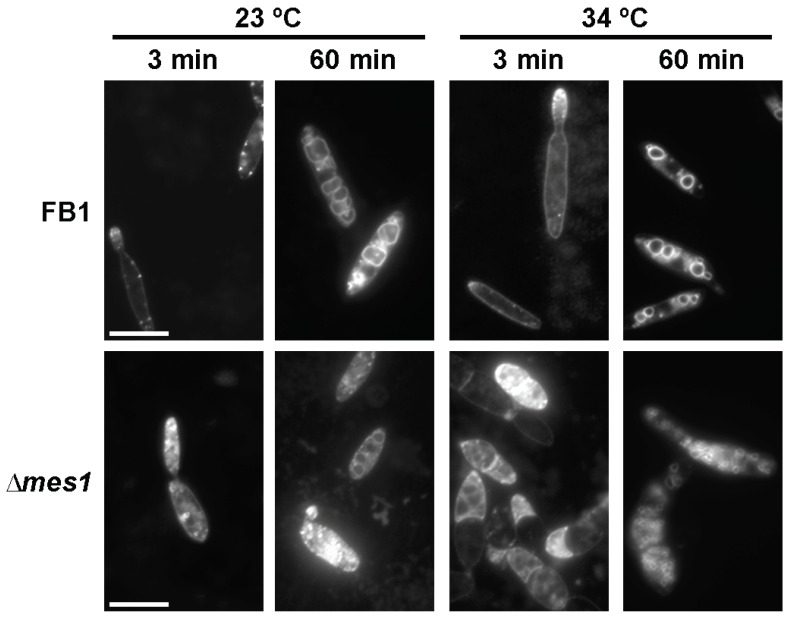
FM4-64 staining of vesicles in *U. maydis* ∆*mes1*. Cells were incubated at the indicated temperature for 4 h and then stained with FM4-64 dye for 3 or 60 min as indicated in the Materials and methods section. Cells were fixed and visualized under the fluorescent microscope. Bar size = 10 µM.

Correct deposition of cell wall material relies on vesicle traffic. As *A. nidulans* and *F. graminearum* ∆*mesA/*∆*mes1* cells showed defects in vesicle organization [11], I used the lipophylic dye FM4-64 in a typical pulse-chase experiment to visualize vacuoles, endosomes and the Spitzenkörper [52]. Wild-type FB1 and *mes1* mutant cells were stained with FM4-64 for 3 min at both a permissive (23 °C) and a restrictive (34 °C) temperature. Wild-type cells showed a bright area of FM4-64 staining localized at the tip of the bud. The dye also stained the plasma membrane and accumulated at defined areas (Figure 5), as previously reported [38,53]. However, in the mutant strain, staining was dispersed at both temperatures. At 34 °C, there was a heterogeneous behaviour of the cells, with some cells showing strong dispersed staining and others showing very faint signals, sometimes displaying disperse aggregates arranged in patches. When the wild-type cells were pulsed with the dye for 2 min and allowed to internalize the dye for 60 min, FM4-64 dye appeared to be associated with the vacuolar membrane. Interestingly, in the mutant strain, only a small portion of the dye reached the vacuolar membrane and showed a perturbed vacuolar structure, especially at 34 °C (Figure 5). Instead of a few big vacuoles, a large number of small vesicles was observed in the mutant strain grown at the restrictive temperature. This suggests defects in endocytosis and vacuolar morphology in the *mes1* deletion strain.

### 3.7. Role of Mes1 in Mating and Plant Infection

**Figure 6:**
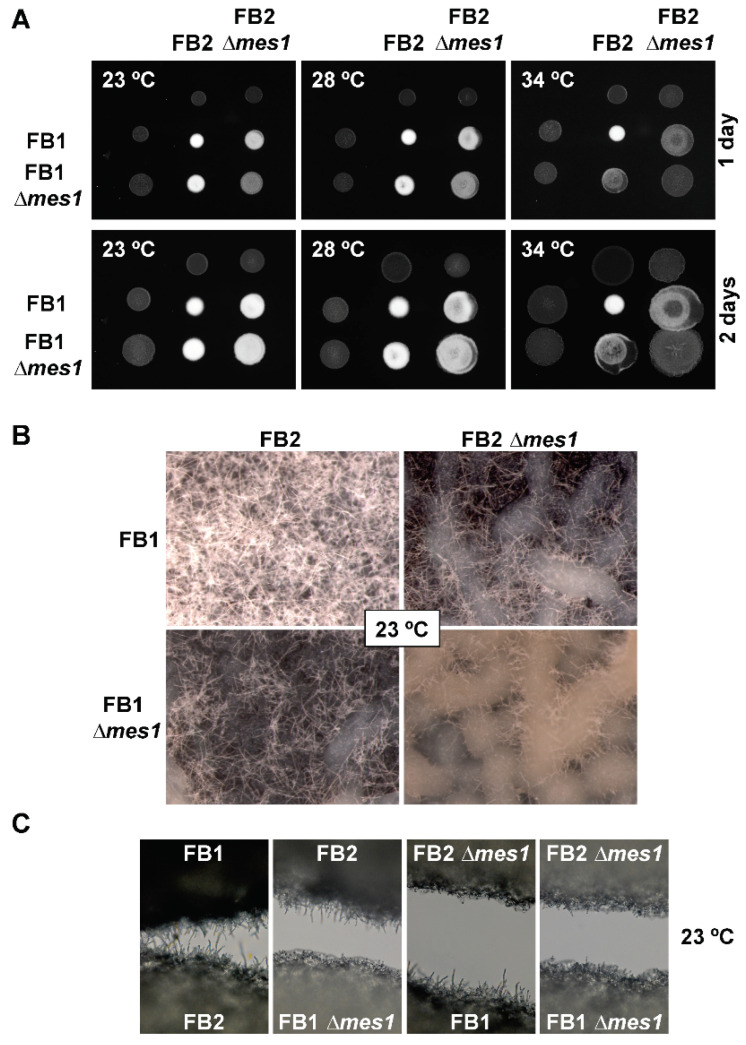
Role of Mes1 in mating. Assays of control strains FB1 × FB2 and ∆*mes1* strains FB1 ∆*mes1* × FB2 ∆*mes1* on PD charcoal-containing agar plates. (**A**) Plates were photographed after one and two days of growth at the indicated temperatures. (**B**) Magnifications of the mating colonies after two days of growth at 23 °C. Photographs taken with a stereomicroscope at 10× show a dense filamentation of the FB1 × FB2 mixture, and reduced filamentation of FB1 × FB2 ∆*mes1* and FB1 ∆*mes1* × FB2. Filamentation of FB1 ∆*mes1* × FB2 ∆*mes1*, although very scarce, is still visible. (**C**) Confrontation assays between all compatible pairs were performed for 20 h at 23 °C, and this shows a reduced filamentation of the ∆*mes1* strains.

For plant infection, two compatible strains must undergo a short period of filamentous growth resulting in the mating process. Then, the infective filament is produced [17]. The requirement of Mes1 during both processes was assessed. For the mating test, compatible FB1 and FB2 ∆*mes1* strains were mixed together on charcoal-containing agar plates and incubated for up to two days. Since the ∆*mes1* phenotype was more dramatic at higher temperatures, then the mating test was carried out at different temperatures (Figure 6). Regardless of the temperature, wild-type FB1 and FB2 strains were able to undergo mating as shown by the presence of a white fuzzy mycelium [54]. When FB1 ∆*mes1* and FB2 ∆*mes1* were co-cultivated on charcoal plates, there was a delay in mating at a low temperature (23 °C), but mutant cells displayed a white fuzzy appearance after two days. Increasing the incubation temperature reduced this white appearance, suggesting defects in the mating process. At 34 °C, mating did not occur even when one of the partners was wild-type. Strikingly, when at least one of the mating partners harboured the deletion mutation, the diameter of the colony was longer than when two wild-type strains were co-cultivated. Mating colonies were visualized under a stereomicroscope (Figure 6B). The patches containing the mating mixture of the two wild-type strains (FB1 and FB2) displayed a dense mat of long filaments, while the mating mixture containing one wild-type and one deletion strain (FB1 × FB2 ∆*mes1*, or FB1 ∆*mes1* × FB2) displayed reduced filamentation. In the patch containing both compatible deletion strains (FB1 ∆*mes1* × FB2 ∆*mes1*), some short filaments could be observed, but both the length and the density of filamentation were reduced compared to the patches containing either one wild-type or both wild-type strains. Similar results were found with confrontation assays (Figure 6C), where deletion strains displayed a drastic reduction in filament length in all possible compatible pairs. Collectively, this suggests that the lack of Mes1 provoked a drastic reduction in filamentation, but it is not completely abolished at 23 °C. The ∆*mes1* short and scarce filaments seem enough to make up for cell fusion and the mating process to occur.

Then, the question was whether short filaments were capable of infecting maize plants. FB1 ∆*mes1* and FB2 ∆*mes1* strains were employed in a virulence test. Because the ∆*mes1* strains are temperature-sensitive, the pathogenicity assays were carried out at the permissive temperature of 25 °C to overpass the growth defects observed in vitro at higher temperatures. Infection with the respective wild-type strains led to tumour production in over 90% of the inoculated plants (Table 1). By contrast, when plants were inoculated with the mutant strains, no symptoms of infection were observed, suggesting that Mes1 is necessary for virulence.

## 4. Discussion

MesA/Mes1 is a fungal protein that was originally identified in the filamentous fungus *A. nidulans* [10] and, later on, it was also characterized in the *F. graminearum* [11]. To date, a role of MesA-like proteins has only been found in filamentous fungi. Here, I characterize the role of Mes1 in a dimorphic fungus, and found evident mutant phenotypes during yeast growth, unlike the deletion of the corresponding homologs in budding or fission yeasts. The results suggest that in *U. maydis* Mes1 is involved in cell wall and plasma membrane integrity, and polarized growth.

Homologous proteins were identified in *S. pombe* and in *S. cerevisiae*. However, the deletion of those genes did not reveal any clear role in morphogenesis or cell wall stability, and only modest defects in length and mating efficiency were found in *S. pombe* [10]. Indeed, MesA/Mes1 homologues from these ascomycete yeasts cluster separately from the rest of the filamentous fungal homologues. Although *U. maydis* yeast cells grow by budding, in the same way as *S. cerevisiae*, *U. maydis* cells are elongated, with a cigar-like shape. Absence of Mes1 produced round cells, more similar to the spherical cells of *S. cerevisiae*. *Cryptococcus neoformans* poses an interesting case, as its homolog clusters together with the *U. maydis* Mes1 but yet, *C. neoformans* displays spherical yeast cells that divide by budding, such as *S. cerevisiae*, and it undergoes filamentous dimorphic transitions, including conjugation tubes similar to those found in *U. maydis* [55].

In addition, in *S. cerevisiae* the distant homolog Avl9 was identified [12]. Phylogenetic studies indicated that MesA/Mes1 and Avl9 belong to a large superfamily of proteins that have an evolutionary relationship with an ancient domain, DENN. The work carried out in scAvl19p uncovered functions in the late secretory pathway and membrane trafficking [12]. Interestingly, the studies performed in *A. nidulans* and *F. graminearum* revealed vacuolar organization defects and the absence of a presumed Spitzenkörper in *mesA*/*mes1* mutants [11]. This study also found defects in the early stages of endocytosis and in the vacuolar organization, even at the permissive temperature, 23 °C. The heavy FM4-64 stain found randomly localized in the cells may have obscured the Spitzenkörper staining. Therefore, in principle, I cannot completely rule out the absence of a Spitzenkörper in the mutant cells (particularly at the permissive temperature, where cells can grow normally). However, the absence of a Spitzenkörper is compatible with the loss of polar growth found at 34 °C.

*mes1* mutants showed an absence of Sep1-GFP at the bud tip, but not at the neck, which is also compatible with the loss of polarized growth, but retaining the capacity of cell division. Interestingly, septin [28] and *mes1* mutants share similar phenotypes, i.e., temperature sensitivity that is remediable with sorbitol, sensitivity to cell wall inhibitors, and similar morphological defects. However, *mes1* mutants also showed additional defects not found in the septin mutants; the ∆*mes1* strain displayed defects in the actin patches and likely in the actin cables, and it is avirulent. Septins act as scaffolds that regulate F-actin interactions, and also gather proteins involved in endocytosis and exocytosis [46]. Septins are essential for the determination of cell shape [46], which is evident in the septin mutants of *U. maydis* [28]. Taken altogether, it is tempting to speculate that Mes1 may interact with septins or contribute to the assembly or stability of the septin scaffolds. Further work is needed to confirm (or reject) this hypothesis. Additionally, Mes1 must play additional roles that explain the other phenotypes, which are more drastic in the *mes1* mutant than in the septin mutants. In *A. nidulans*, MesA is required for the recruitment of the formin SepA and the proper assembly of actin cables at the polarization sites [10], which is in agreement with the loss of Myo5-GFP localization (and presumably actin cables at the tip of the bud) in a *mes1* deletion strain in *U. maydis*. This work also found that Mes1 is also required for the assembly of actin cortical patches, endocytosis and proper vacuolar organization. Altogether, these defects can explain the loss of polarized growth and the more drastic phenotype compared to the septin mutants.

There was an absolute requirement of Mes1 for virulence. ∆*mes1* strains can undergo mating at a low temperature, although the mating experiments suggested that the mating is not very efficient. Cells depleted of Mes1 are incapable of achieving the strong *b*-dependent polarized growth required for the formation of the infective filament. Then, the defects in virulence of the ∆*mes1* strain could be due to a combination of the reduction in polarized growth, instability of the cell wall, and defects in endocytosis and vacuolar organization. These results are interesting, because MesA/Mes1 could become a new target for the development of novel antifungal drugs aiming at both animal and plant pathogenic filamentous fungi and dimorphic fungi, in which the filamentous growth form is required for virulence.

## Figures and Tables

**Table 1:** Pathogenicity assays.

		Tumour Formation ^a^	
Inoculum	Genotype	Total	Percentage
FB1 × FB2	*a1 b1* × *a2 b2*	32/35	91
UMD12 × UMD16	*a1 b1* ∆*mes1 × a2 b2* ∆*mes1*	0/47 ^b^	0

^a^ The pathogenicity assays were performed at 25 °C. ^b^ Only chlorosis was observed.

## Data Availability

Not applicable.

## Supplementary Materials

The following supporting information can be downloaded at: https://www.mdpi.com/article/10.3390/jof8080759/s1. Table S1. Strains used in this study. Table S2. Primers used in this study. Figure S1. Growth curves of FB1 and FB1 ∆*mes1* on YPD solid media. Figure S2. Supplementation of liquid media with sorbitol did not remediate the polarization defects of the ∆*mes1* strain. Figure S3. The conditional allele *mes1^nar^* recapitulates the ∆*mes1* deletion phenotype. Figure S4. Lack of *mes1* hinders filamentation at 23 °C.
