# Supplementary material for: The Fungal Protein Mes1 Is Required for Morphogenesis and Virulence in the Dimorphic Phytopathogen Ustilago maydis
Source: J Fungi (Basel). 2022 Jul 22;8(8):759. doi: 10.3390/jof8080759 (PMC9331856; doi:10.3390/jof8080759)
Supplement: Supplementary file 1 [file jof-08-00759-s001.zip › jof-1739682-supplementary.pdf]

Table S1. *U. maydis* strains used in this study.

Strain	Relevant genotype	Reference
FB1	*a1 b1*	[21]
FB2	*a2 b2*	[21]
AB31	$a2\ P_{crg}{:}bW2\ P_{crg}{:}bE1$	[23]
AB33	$a2\ P_{nar1}{:}bW2\ P_{nar1}{:}bE1$	[23]
UMD4	$a1\ b1\ mes1^{nar}$	This study
UMD12	*a1 b1 Δmes1*	This study
UMD16	*a2 b2 Δmes1*	This study
UMP71	*a1 b1 fim1-gfp*	[40]
UMD9	$a2\ P_{nar1}{:}bW2\ P_{nar1}{:}bE1\ \Delta mes1$	This study
UMD7	$a2\ P_{crg}{:}bW2\ P_{crg}{:}bE1\ mes1^{nar}$	This study
UMD17	$a2\ P_{crg}{:}bW2\ P_{crg}{:}bE1\ mes1^{nar}$ *fim1-gfp*	This study
UMD18	$a2\ P_{crg}{:}bW2\ P_{crg}{:}bE1\ mes1^{nar}$ *myo5-gfp*	This study
UMD19	$a2\ P_{crg}{:}bW2\ P_{crg}{:}bE1\ mes1^{nar}$ *sep1-gfp*	This study

Table S2. Primers used in this study.

mes1-2	5'-CGGGGTACCACTCGATGATGTCACTGAAAG-3'
mes1-4	5'-CGCGGATCCATCACCGAAGCTTCCAACGAA-3'
mes1-5	5'-CGGGGTACCGGCAACGTGACTGGCTGGCTG-3'
mes1-8	5'-CCGCTTAAGCTTGATACCACTTTTGACAGT-3'
mes1-9	5'-AAGGCCTAGATGGCCGATTTCGAGCAGGGCGGCGGGCTT-3'
mes1-10	5'-AAGGCCTGAGTGGCCTAGCATACGATTCACGATTGTCGC-3'
mes1-11	5'-TCCATCGCTTCGTCTTTTGCTTCCGC-3'

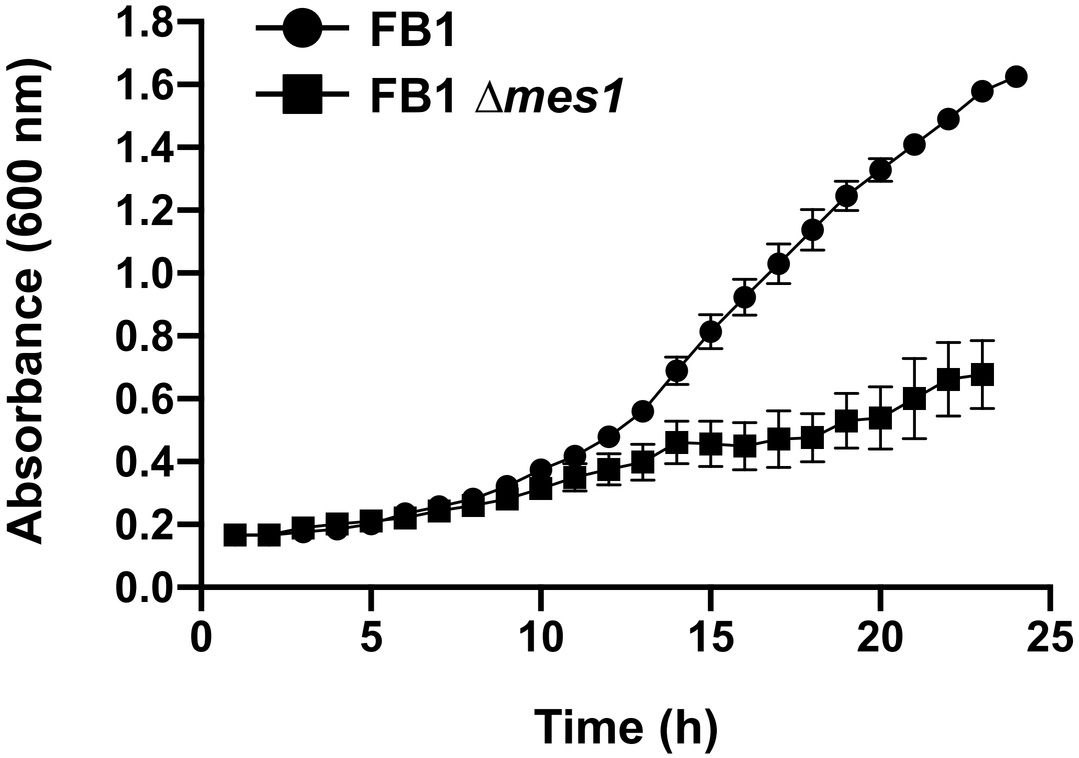

Figure S1. Growth curve of FB1 and FB1 Δ*mes1* strains on YPD solid media. Strains were grown in liquid media overnight and cultures were diluted to a final OD_{600nm} of 0.1. 30 µl were employed to inoculate YPD solid media in a 96-well plate and growth was monitored every hour for 24 h by using a plate reader as previously reported .

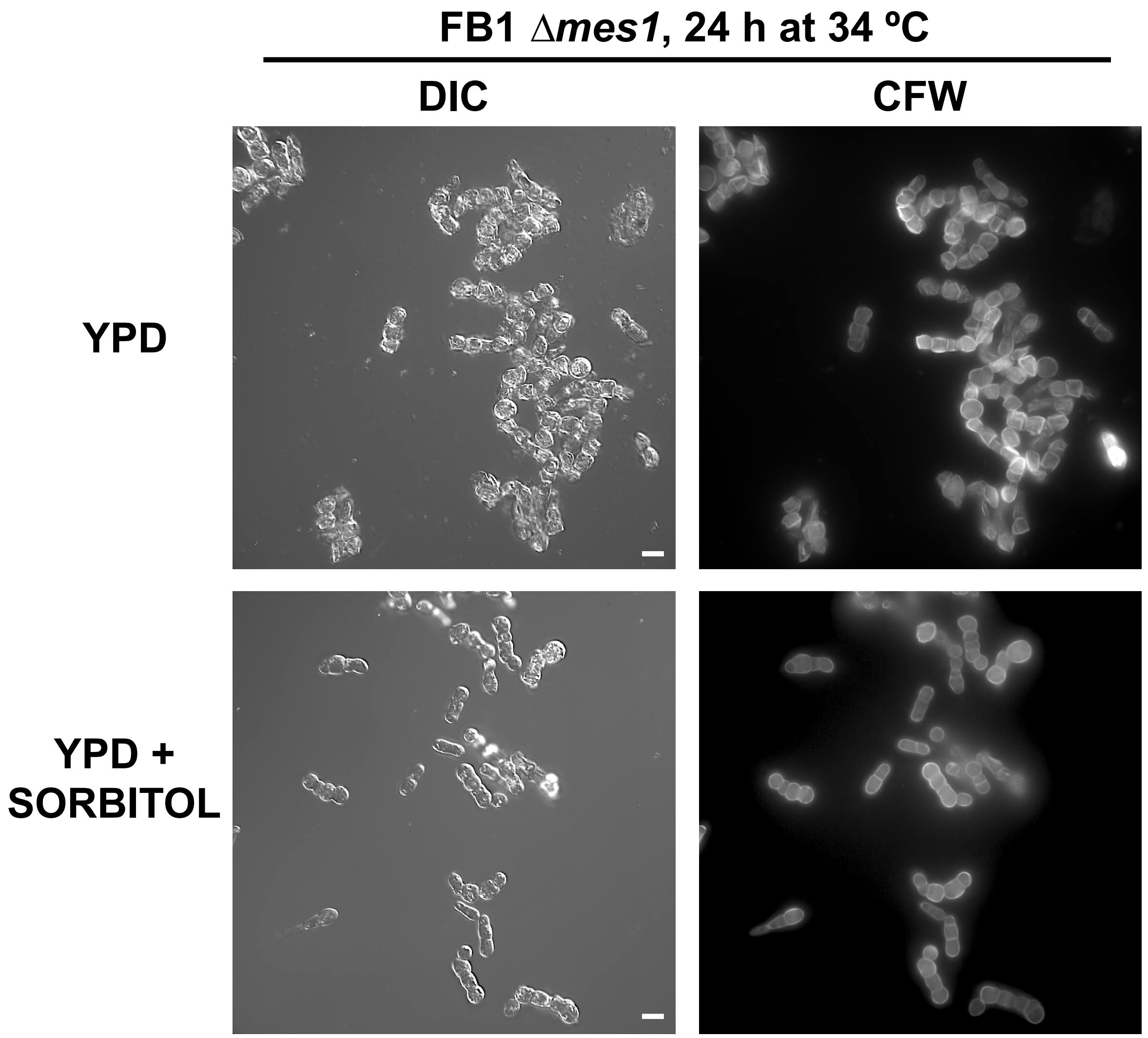

Figure S2. Supplementation of liquid media with sorbitol did not remediate the polarization defects of the ∆*mes1* strain. FB1 and FB1 ∆*mes1* strains were grown overnight in YPD at 23 ºC, diluted into fresh YPD or YPD + 1 M sorbitol and incubated at 34 ºC for 24 h. Cells were stained with calcofluor and photographed under the fluorescent microscope. Bar size = 10 µm.

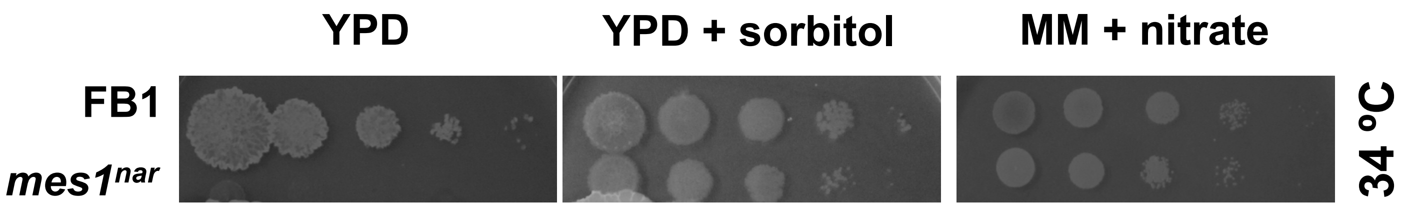

Figure S3. The conditional allele *mes1^nar^* recapitulates the ∆*mes1* deletion phenotype. 10-fold serial dilutions of FB1 and FB1 *mes1^nar^* strains were spotted on YPD plates with or without 1 M sorbitol, or on plates containing glucose minimal medium with nitrate to repress (YPD) or to induce (MM+nitrate) *mes1* expression. Plates incubated at 34 ºC and photographed.

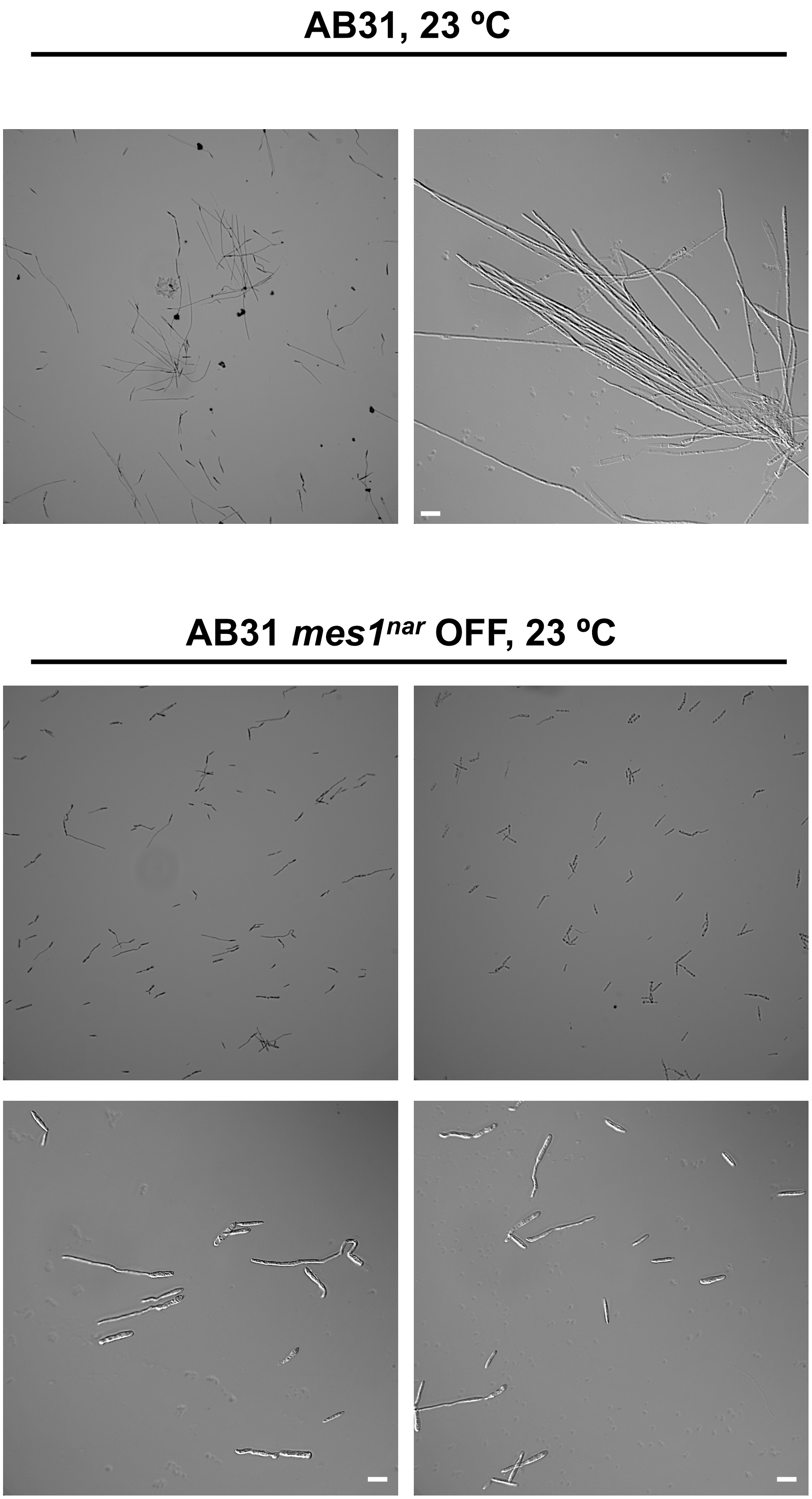

Figure S4. Lack of *mes1* hinders filamentation at 23 ºC. AB31 and AB31 *mes1*^{nar} were grown in YPD media at 23 ºC. Cells were washed and incubated in CMA media overnight to induce *b*-dependent filamentation. Bar size = 10 µm.
